# Shear Wave Elastography Assessment of Achilles Tendon Stiffness in Asymptomatic Patients with Psoriatic Arthritis

**DOI:** 10.3390/diagnostics16050742

**Published:** 2026-03-02

**Authors:** Veysel Burulday, Nurullah Dag, Aysun Gunduz Uslu, Servet Yolbas

**Affiliations:** 1 Department of Radiology, Faculty of Medicine, Inonu University, Malatya 44280, Türkiye; veysel.burulday@inonu.edu.tr (V.B.); gunduzaysun4@gmail.com (A.G.U.); 2 Department of Rheumatology, Faculty of Medicine, Inonu University, Malatya 44280, Türkiye; servet.yolbas@inonu.edu.tr

**Keywords:** psoriatic arthritis, achilles tendon, tendon stiffness, shear wave elastography, ultrasonography

## Abstract

**Objectives**: We aimed to evaluate Achilles tendon stiffness characteristics in asymptomatic patients with psoriatic arthritis (PsA) using shear wave elastography (SWE). **Methods**: In this prospective case–control study, 34 asymptomatic PsA patients and 34 age- and sex-matched healthy controls underwent bilateral Achilles tendon evaluation with grayscale ultrasonography and SWE. Tendon thickness was measured 3 cm proximal to the calcaneal insertion. Shear-wave velocity (m/s) and Young’s modulus (kPa) were obtained under standardized acquisition conditions, including five-star motion stability and ≥90% reliability. **Results**: Achilles tendon morphology and thickness did not differ between PsA patients and controls (*p* > 0.05). In contrast, SWE demonstrated higher tendon stiffness in the PsA group. Mean shear-wave velocity was significantly greater in PsA patients for both the left (4.89 ± 2.52 m/s vs. 3.23 ± 0.41 m/s; *p* < 0.001) and right tendons (4.88 ± 1.94 m/s vs. 3.12 ± 0.30 m/s; *p* < 0.001), with corresponding increases in Young’s modulus (all *p* < 0.001). SWE demonstrated good group discrimination, with shear-wave velocity achieving AUC values of up to 0.90 in differentiating PsA patients from healthy controls. **Conclusions**: SWE may reflect biomechanical tendon alterations in PsA, even in the absence of clinical symptoms, and may serve as a complementary imaging tool in the assessment of tendon involvement.

## 1. Introduction

Psoriatic arthritis (PsA) is a chronic immune-mediated inflammatory disease associated with psoriasis and characterized by a heterogeneous spectrum of musculoskeletal manifestations, including peripheral and axial arthritis, dactylitis, and entheseal involvement [1,2,3]. Among individuals with psoriasis, PsA affects a substantial proportion of patients worldwide and is frequently underdiagnosed, with diagnostic delays exceeding two years in many cases [4,5]. Such delays are clinically relevant, as persistent inflammatory activity may lead to irreversible structural damage, functional impairment, and reduced quality of life.

Enthesitis, defined as inflammation at the tendon or ligament insertion site, is considered a hallmark feature of PsA and is closely associated with disease activity and overall disease burden [6,7]. The Achilles tendon is one of the most commonly involved entheseal sites and has a major role in gait mechanics and load transmission [8]. However, physical examination alone has limited sensitivity for detecting early or subtle musculoskeletal involvement, particularly in the absence of pain or tenderness [9]. Imaging modalities therefore play a crucial role in the assessment of entheseal and peri-entheseal pathology.

Ultrasonography, including grayscale and power Doppler techniques, is more sensitive than clinical examination for identifying entheseal abnormalities and may reveal subclinical changes in patients with psoriasis or PsA [9,10]. Magnetic resonance imaging (MRI) provides complementary information, particularly regarding bone marrow edema and peri-entheseal inflammation, but its routine use is limited by cost, availability, and spatial resolution for superficial structures [11]. Despite these advances, conventional imaging methods primarily capture morphological or vascular changes and may fail to detect early biomechanical alterations within tendon tissue.

Beyond the enthesis, chronic inflammatory conditions such as psoriatic arthritis may also induce biomechanical changes in adjacent tendon tissue, even before overt clinical involvement. The Achilles tendon represents a continuous functional unit in which inflammatory, mechanical, and reparative processes may extend beyond the insertional region into the tendon mid-portion [2,3,4,5,6,7]. Evaluation of tendon mechanical properties may therefore provide additional insights into early musculoskeletal involvement that are not apparent on standard grayscale imaging.

Shear wave elastography (SWE) is an advanced ultrasonographic technique that quantitatively assesses tissue stiffness by measuring shear wave propagation velocity, offering an objective and reproducible evaluation of tendon mechanical properties [12]. Unlike conventional ultrasonography, SWE does not primarily rely on morphological alterations but reflects composite changes in tissue elasticity that may be influenced by inflammation, fibrosis, or chronic mechanical loading [13,14]. However, SWE does not allow clear differentiation between active inflammatory processes and chronic structural remodeling, and stiffness alterations should therefore be interpreted as nonspecific biomechanical changes rather than direct markers of inflammatory activity.

Although previous studies have investigated SWE in symptomatic tendon disorders and inflammatory arthropathies, data regarding asymptomatic patients with PsA remain limited [14,15]. In particular, the potential of SWE to detect biomechanical tendon alterations in the absence of clinical or grayscale ultrasonographic abnormalities has not been fully elucidated. Therefore, the aim of this study was to evaluate Achilles tendon stiffness using shear wave elastography in asymptomatic patients with psoriatic arthritis, focusing on biomechanical alterations beyond overt clinical enthesitis.

## 2. Materials and Methods

### 2.1. Study Design and Ethical Approval

This prospective, cross-sectional, case–control study was conducted at a tertiary care center between October 2024 and February 2025. The study protocol was approved by the Institutional Review Board of Inonu University (approval no. 2024/143). Written informed consent was obtained from all participants prior to enrollment, and the study was conducted in accordance with the Declaration of Helsinki and institutional regulatory standards.

### 2.2. Patient Selection

The study included 34 asymptomatic adult patients with psoriatic arthritis (PsA) and 34 age- and sex-matched healthy controls. The diagnosis of PsA was confirmed by an experienced rheumatologist according to the Classification Criteria for Psoriatic Arthritis [16].

Inclusion criteria for the PsA group were age between 18 and 65 years, a confirmed diagnosis of PsA for at least two years, and the absence of pain, tenderness, swelling, or functional limitation in the ankle or Achilles tendon region at the time of examination.

Exclusion criteria for both groups included diabetes mellitus, rheumatoid arthritis, ankylosing spondylitis, inflammatory bowel disease, history of ankle trauma or known Achilles tendinopathy, prior ankle surgery within the preceding six months, and current or previous systemic corticosteroid or biologic therapy. Patients receiving systemic corticosteroid or biologic therapy were excluded to minimize potential treatment-related effects on tendon mechanical properties and to ensure a more homogeneous assessment of disease-related biomechanical changes.

The control group consisted of healthy volunteers with no history of musculoskeletal or rheumatologic disease and no clinical symptoms related to the Achilles tendon.

### 2.3. Ultrasonography and Shear Wave Elastography Protocol

All ultrasonographic and shear wave elastography (SWE) examinations were performed using a single ultrasound system (Resona I9, Mindray, Shenzhen, China) equipped with a linear-array transducer (L14–3Ws). All scans were conducted by one radiologist with five years of experience in musculoskeletal ultrasonography to ensure intraobserver consistency, and identical technical parameters were used throughout the study.

Participants were positioned prone on the examination table with their feet hanging freely beyond the edge to maintain a neutral ankle position and minimize tendon tension. A generous amount of coupling gel was applied, and minimal transducer pressure was used to avoid compression-related artifacts.

**Figure 1:**
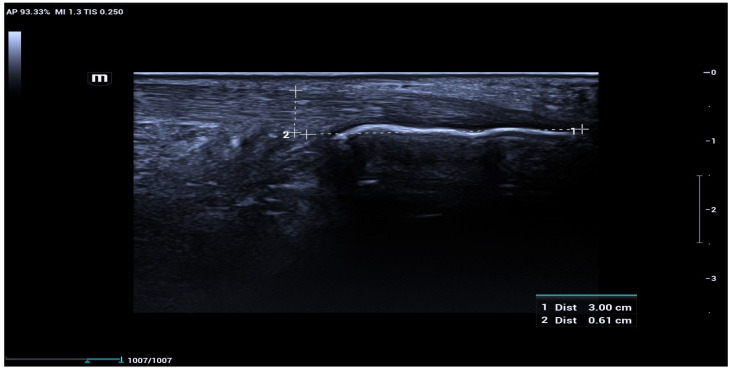
Measurement of Achilles tendon thickness. Longitudinal grayscale ultrasonographic image demonstrating measurement of Achilles tendon thickness performed 3 cm proximal to the calcaneal insertion. The measurement was obtained with the patient in the prone position and the ankle in a neutral position, ensuring minimal transducer pressure.

Grayscale ultrasonography of the Achilles tendon (mid-portion) was performed in both longitudinal and axial planes to evaluate tendon morphology and echotexture, and tendon thickness was measured in the longitudinal plane at a standardized location 3 cm proximal to the calcaneal insertion, corresponding to the mid-portion of the Achilles tendon (Figure 1).

**Figure 2:**
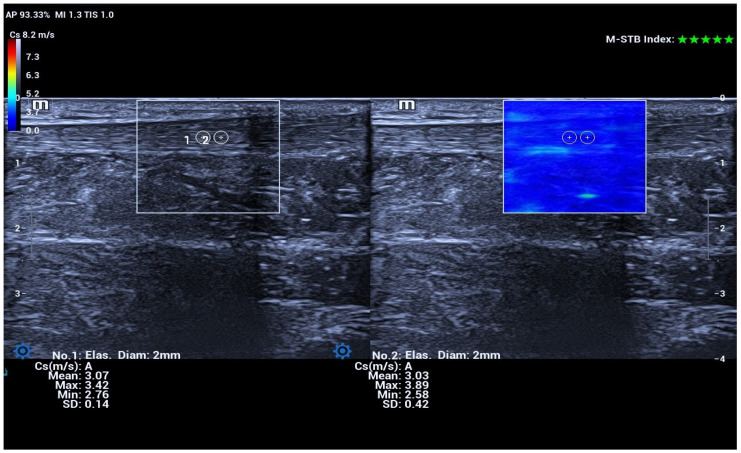
Shear wave elastography of the Achilles tendon. Longitudinal shear wave elastography image of the Achilles tendon showing placement of the region of interest within the tendon.

For SWE acquisition, three longitudinal image sections were obtained for each tendon, with two repeated measurements per section. A circular region of interest (ROI) with a diameter of 3 mm was placed carefully within the tendon substance, avoiding the paratenon, vascular structures, and visible artifacts. The measurement range was set to 0–8.2 m/s for shear-wave velocity and 0–200 kPa for Young’s modulus. SWE measurements were obtained under standardized acquisition conditions, including a five-star Motion Stability Index and a reliability index ≥90%, with ROI placement at the mid-portion of the Achilles tendon [17,18] (Figure 2).

Measurements were intentionally performed at the mid-portion of the Achilles tendon to ensure reproducibility and avoid calcaneal acoustic artifacts.

Because shear-wave velocity (m/s) and Young’s modulus (kPa) may vary depending on tissue anisotropy and vendor-specific reconstruction algorithms, shear-wave velocity was considered the primary stiffness parameter, whereas Young’s modulus was used as a complementary secondary metric.

### 2.4. Statistical Analysis

Statistical analyses were performed using IBM SPSS Statistics version 26.0 (IBM Corp., Armonk, NY, USA). Continuous variables were summarized as mean ± standard deviation and median (minimum–maximum), while categorical variables were expressed as frequency and percentage. Normality of data distribution was assessed using the Shapiro–Wilk test.

For between-group comparisons, the independent samples *t*-test was used for normally distributed variables, and the Mann–Whitney U test was applied for non-normally distributed variables. Categorical variables were compared using the continuity correction chi-square test. Correlations were assessed using Pearson or Spearman correlation coefficients, as appropriate.

For side-based analyses, right and left Achilles tendons were evaluated separately; for ROC analysis, a single representative value (maximum shear-wave velocity per patient) was used to avoid violation of statistical independence.

ROC analysis was performed to assess the ability of SWE parameters to differentiate patients with PsA from healthy controls. The area under the curve (AUC) with 95% confidence intervals, optimal cut-off values derived using the Youden index, sensitivity, specificity, positive predictive value, and negative predictive value were reported. A *p* value < 0.05 was considered statistically significant.

An a priori sample size calculation was conducted using G*Power software (version 3.1). Based on an assumed effect size of 0.72, a two-sided alpha level of 0.05, and a statistical power of 80%, the minimum required sample size was determined to be 32 participants per group. The final study cohort met this requirement.

## 3. Results

### 3.1. Participant Characteristics

A total of 68 participants were included in the study, comprising 34 asymptomatic patients with PsA and 34 age- and sex-matched healthy controls. Sex distribution was comparable between groups, with females representing 76.5% (*n* = 26) of the PsA group and 73.5% (*n* = 25) of the control group (*p* = 1.000). There were no significant differences between groups regarding age, height, weight, or body mass index (all *p* > 0.05). The mean age was 48.21 ± 10.07 years in the PsA group and 47.91 ± 10.14 years in the control group. These results indicate that the study groups were demographically and anthropometrically well matched (Table 1).

### 3.2. Achilles Tendon Thickness and Stiffness Measurements

There was no significant difference in Achilles tendon thickness between patients with PsA and healthy controls. The mean thickness of the left Achilles tendon was 0.46 ± 0.09 cm in the PsA group and 0.47 ± 0.09 cm in the control group (*p* = 0.708), whereas the corresponding values for the right tendon were 0.47 ± 0.09 cm and 0.48 ± 0.08 cm, respectively (*p* = 0.530).

In contrast, SWE demonstrated significantly increased tendon stiffness in PsA patients compared with controls. The mean shear-wave velocity for the left Achilles tendon was 4.89 ± 2.52 m/s in the PsA group and 3.23 ± 0.41 m/s in the control group (*p* < 0.001), corresponding to mean elasticity values of 35.68 ± 46.48 kPa and 10.74 ± 2.70 kPa, respectively (*p* < 0.001). For the right Achilles tendon, mean shear-wave velocity was 4.88 ± 1.94 m/s in the PsA group and 3.12 ± 0.30 m/s in controls (*p* < 0.001), with corresponding elasticity values of 31.90 ± 33.98 kPa and 10.00 ± 1.95 kPa (*p* < 0.001). These findings indicate markedly increased tendon stiffness in PsA patients relative to healthy controls (Table 2).

### 3.3. Discriminative Ability of SWE Parameters

To evaluate the ability of SWE parameters to differentiate patients with psoriatic arthritis from healthy controls, ROC analysis was performed. Achilles tendon thickness demonstrated limited discriminative ability, with an AUC of 0.500 (95% CI: 0.377–0.624, *p* = 0.995) for the left side and 0.518 (95% CI: 0.393–0.641, *p* = 0.803) for the right side.

**Figure 3:**
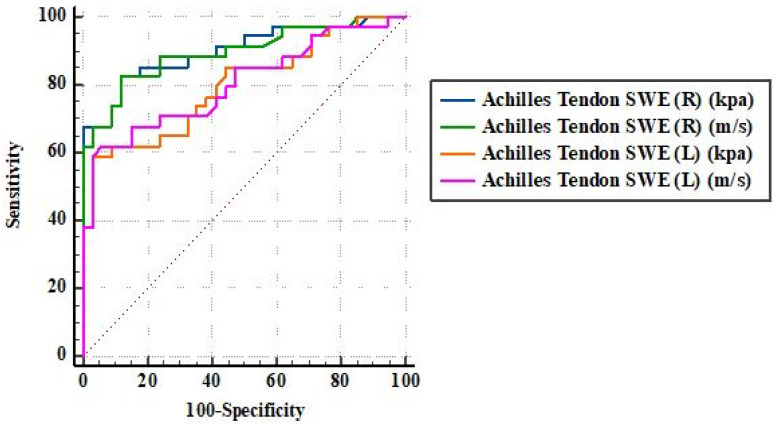
ROC curves illustrating the discriminative performance of shear-wave velocity and Young’s modulus values in differentiating patients with psoriatic arthritis from healthy controls.

In contrast, SWE parameters exhibited excellent discriminative performance between the PsA and control groups. For the left Achilles tendon, shear-wave velocity achieved an AUC of 0.804 (95% CI: 0.689–0.890, *p* < 0.001) with 61.76% sensitivity and 94.12% specificity, while the corresponding Young’s modulus yielded an AUC of 0.798 (95% CI: 0.683–0.886, *p* < 0.001). For the right Achilles tendon, SWE velocity demonstrated an AUC of 0.896 (95% CI: 0.798–0.957, *p* < 0.001) with 82.35% sensitivity and 88.24% specificity, whereas Young’s modulus showed a similar diagnostic performance with an AUC of 0.900 (95% CI: 0.803–0.960, *p* < 0.001) (Table 3, Figure 3).

Although Achilles tendon thickness values showed no statistically significant differences between groups, SWE-derived stiffness parameters demonstrated superior discriminative performance for distinguishing asymptomatic PsA patients from healthy controls.

### 3.4. Unilateral Versus Bilateral Involvement

Among patients with psoriatic arthritis, unilateral Achilles tendon involvement was observed in 8 patients, whereas bilateral involvement was present in 26 patients. Comparison between these two subgroups demonstrated no significant difference in Achilles tendon thickness measurements on either side (all *p* > 0.05). In contrast, SWE measurements revealed significantly higher stiffness values in patients with unilateral involvement. Both shear-wave velocity and Young’s modulus values were significantly increased in the unilateral involvement group for the left Achilles tendon (*p* = 0.022 and *p* = 0.017, respectively) as well as for the right Achilles tendon (*p* = 0.035 and *p* = 0.028, respectively) (Table 4).

## 4. Discussion

In this study, patients with PsA who exhibited no clinical signs of enthesitis demonstrated significantly increased Achilles tendon stiffness compared with healthy controls. Despite normal tendon thickness and unremarkable grayscale ultrasonography, SWE revealed marked alterations in biomechanical properties, suggesting that SWE may reflect early biomechanical tendon alterations that precede overt entheseal abnormalities. While previous studies on symptomatic PsA have focused on tendon thickening, erosions, or Doppler-active inflammation, evidence regarding subclinical changes remains limited. The present findings therefore contribute to growing data supporting the sensitivity of SWE in identifying early entheseal alterations in PsA. From a clinical perspective, detection of subclinical entheseal alterations may support earlier clinical awareness and closer monitoring of patients who are otherwise considered clinically inactive; however, the cross-sectional design precludes any inference regarding predictive or prognostic value. This may be particularly relevant in patients with longstanding disease but minimal symptoms.

Conventional grayscale ultrasonography is widely used for detecting entheseal abnormalities, including tendon thickening, calcifications, enthesophytes, erosions, and Doppler hyperemia [15]. Subclinical abnormalities frequently exceed those identified on physical examination, underscoring the utility of imaging in early disease detection [19,20,21]. MRI offers complementary diagnostic value, particularly for identifying intratendinous edema, peritendinous inflammation, and bone marrow edema [22,23]. However, these modalities primarily capture morphological and vascular features. SWE provides additional value by quantifying the mechanical properties of the tendon, thereby offering improved sensitivity to early inflammatory or fibrotic changes [24,25]. Increased stiffness likely reflects chronic low-grade inflammation or early fibrotic remodelling rather than acute inflammatory oedema.

The diagnostic potential of SWE in musculoskeletal imaging has been demonstrated in various clinical scenarios. Seth et al. [26] highlighted its relationship with tendon integrity, fatty infiltration, and functional outcomes in rotator cuff disorders, while Steiner et al. [27] showed that SWE reliably differentiates normal from tendinopathic tissue in an ex vivo tendon model. These observations support the capacity of SWE to identify microstructural deterioration not evident on grayscale imaging. Findings from PsA cohorts further suggest that SWE measurements may vary according to disease activity. Saha et al. [28] reported that post-exercise SWE values in patients treated with IL-17 inhibitors approached those of healthy controls, implying a modulatory effect of cytokine-targeted therapy. Conversely, studies in symptomatic enthesitis have typically identified reduced elasticity, reflecting edema, inflammatory infiltration, and increased vascular permeability. The increased stiffness observed in our asymptomatic cohort likely reflects chronic low-grade inflammation or early fibrotic remodeling, rather than acute inflammatory edema, which has been associated with reduced elasticity in clinically symptomatic enthesitis. The negative correlation between SWE values and clinical enthesitis scores reported by Guillen et al. [29] suggests that SWE may detect both inflammatory and chronic structural changes depending on disease phase. The relatively wide dispersion of SWE values observed in the PsA group likely reflects biological heterogeneity of subclinical tendon involvement. Variability in disease duration, subclinical inflammatory burden, and individual biomechanical factors may contribute to this finding. Importantly, no extreme outliers requiring exclusion were identified, supporting the overall robustness of the measurements.

The biological mechanisms underlying Achilles tendon involvement in PsA provide a plausible explanation for the increased SWE values observed in this study. Enthesitis is driven by pro-inflammatory cytokines, particularly TNF-α and IL-17, which promote immune cell infiltration, neovascularization, and local edema [30,31]. Over time, chronic or recurrent inflammation may trigger fibrotic remodeling, characterized by collagen accumulation and reduced tissue compliance—changes that would be expected to increase tendon stiffness on elastography. The elevated SWE values in our asymptomatic cohort may therefore reflect low-grade chronic inflammation or fibrosis rather than acute inflammatory edema. This interpretation aligns with the hypothesis proposed by Saha et al. [28], suggesting that higher stiffness values may indicate a transition from active inflammation to reparative or fibrotic remodeling.

Early identification of subclinical enthesitis has meaningful clinical implications, as timely recognition of mechanical changes may prevent progression to symptomatic disease, functional impairment, or tendon damage [2,5]. SWE offers objective, quantitative, and reproducible assessments that complement routine ultrasonographic evaluation. Bilateral examination in this study provided insight into the symmetry of entheseal involvement, and the standardized single-operator protocol strengthened internal validity by reducing technical variability. SWE may therefore contribute to a more individualized and preventive management strategy in PsA.

Patients with unilateral involvement demonstrated higher tendon stiffness; however, given the relatively small size of this subgroup, these findings should be interpreted with caution. This observation may suggest a possible association with focal mechanical loading, but it should be considered exploratory and requires confirmation in larger cohorts. Mechanical stress has been shown to interact with inflammatory pathways in psoriatic arthritis, potentially resulting in localized stiffness changes before bilateral or clinically apparent involvement develops [6,11].

This study has limitations. The lack of correlation with complementary diagnostic tools—such as MRI, biomarker analyses, or histopathology—precludes direct pathophysiologic validation of SWE findings. The absence of longitudinal follow-up limits the ability to determine whether increased tendon stiffness predicts future symptomatic enthesitis or structural deterioration. Treatment heterogeneity represents a potential confounder, and all examinations were performed by a single radiologist prevented assessment of interobserver reproducibility. Although therapeutic implications were not evaluated in this study, increased tendon stiffness may represent a targetable disease domain in future studies assessing treatment response or progression to symptomatic enthesitis. No imaging or histopathological gold standard was available; therefore, the ROC findings should be interpreted as reflecting group discrimination rather than true diagnostic accuracy. These results primarily demonstrate the ability of SWE parameters to differentiate study groups and should not be considered definitive diagnostic performance metrics. The exclusion of patients receiving systemic corticosteroid or biologic therapy may limit the generalizability of the findings to the broader PsA population; however, this approach was intentionally adopted to reduce treatment-related confounding in SWE measurements. In addition, the unilateral involvement subgroup was relatively small, which limits the robustness of subgroup comparisons. Therefore, these findings should be interpreted cautiously and considered hypothesis-generating until validated in larger studies. Larger, multicenter, longitudinal studies integrating multimodal imaging and clinical outcomes are warranted to confirm its diagnostic and prognostic utility.

## 5. Conclusions

Asymptomatic patients with psoriatic arthritis may exhibit increased Achilles tendon stiffness despite normal grayscale ultrasonography, suggesting the presence of subclinical biomechanical tendon alterations. SWE appears to be more sensitive than conventional imaging in detecting these early biomechanical alterations. SWE should be regarded as a complementary imaging tool rather than a replacement for clinical examination or conventional ultrasonography. Overall, SWE may represent a useful adjunctive modality for the early identification of biomechanical tendon alterations in psoriatic arthritis, alongside established clinical and imaging assessments.

## Figures and Tables

**Table 1:** Demographic data of the control and patient groups.

	Group				*p* Value
	Control (*n* = 34)		Patients (*n* = 34)		
	Count	Percent (%)	Count	Percent (%)	
Female	25	73.5%	26	76.5%	1.000 *
Male	9	26.5%	8	23.5%	
	**Mean ± SD**	**Median (IQR)**	**Mean ± SD**	**Median (IQR)**	
Age (years)	47.91 ± 10.14	46.5 (42.7–51.0)	48.21 ± 10.07	49.5 (42.7–56.0)	0.905 **
Height (cm)	163.97 ± 6.56	163.5 (160–165)	162.94 ± 6.54	162 (158–166)	0.490 ***
Weight (kg)	71.38 ± 11.01	70 (65–77)	70.79 ± 9.05	69.5 (64–75)	0.839 ***
BMI (kg/m^2^)	26.5 ± 3.24	25.9 (24.9–29.3)	26.61 ± 2.39	26.27 (24.6–29.2)	0.883 **

BMI: Body Mass Index; SD: Standard Deviation; *: Continuity Correction Chi-Square test; **: Independent Sample *t*-test; ***: Mann–Whitney U test.

**Table 2:** Comparison of Achilles tendon elasticity values and Achilles tendon thickness values between patient and control groups.

	Group				*p* Value
	Control (*n* = 34)		Patients (*n* = 34)		
	Mean ± SD	Median (IQR)	Mean ± SD	Median (IQR)	
Achilles Tendon Thickness (L) (cm)	0.47 ± 0.09	0.45 (0.42–0.50)	0.46 ± 0.09	0.48 (0.38–0.53)	0.708 *
Achilles Tendon SWE (L) (m/s)	3.23 ± 0.41	3.17 (2.86–3.58)	4.89 ± 2.52	3.91 (3.36–4.79)	<0.001 **
Achilles Tendon SWE (L) (kPa)	10.74 ± 2.7	10.30 (8.45–12.97)	35.68 ± 46.48	15.46 (11.68–28.87)	<0.001 **
Achilles Tendon Thickness (R) (cm)	0.48 ± 0.08	0.46 (0.43–0.52)	0.47 ± 0.09	0.47 (0.41–0.52)	0.530 *
Achilles Tendon SWE (R) (m/s)	3.12 ± 0.3	3.11 (2.88–3.31)	4.88±1.94	4.31 (3.52–5.34)	<0.001 **
Achilles Tendon SWE (R) (kPa)	10 ± 1.95	10.13 (8.50–11.12)	31.9±33.98	18.78 (12.61–33.78)	<0.001 **

R: Right; L: Left; SWE: Shear wave velocity; SD: Standard Deviation; *: Independent Sample *t*-test; **: Mann–Whitney U test.

**Table 3:** Discriminative Performance of Achilles Tendon Thickness and SWE Parameters for Differentiating Patients and Controls.

Variables	Cut-Off	Sensitivity (%)	Specificity (%)	PPV (%)	NPV (%)	AUC (95%CI)	*p* Value
Achilles Tendon SWE (R) (m/s)	>3.48	82.3	88.24	87.5	83.3	0.896 (0.798–0.957)	<0.001
Achilles Tendon SWE (R) (kPa)	>12.14	82.3	88.2	87.5	83.3	0.900 (0.803–0.960)	<0.001
Achilles Tendon SWE (L) (m/s)	>3.85	61.7	94.1	91.3	71.1	0.804 (0.689–0.890)	<0.001
Achilles Tendon SWE (L) (kPa)	>14.97	58.8	97.0	95.2	70.2	0.798 (0.683–0.886)	<0.001

**Table 4:** Comparison of Achilles tendon thickness and shear wave elastography parameters between unilateral and bilateral involvement in patients with psoriatic arthritis.

	Involvement				*p* Value
	Unilateral Involvement (*n* = 8)		Bilateral Involvement (*n* = 26)		
	Mean ± SD	Median (IQR)	Mean ± SD	Median (IQR)	
Achilles Tendon Thickness (L) (cm)	0.42 ± 0.09	0.44 (0.33–0.49)	0.48 ± 0.08	0.49 (0.41–0.53)	0.120 *
Achilles Tendon SWE (L) (m/s)	7.02 ± 3.48	6.16 (3.89–10.64)	4.24 ± 1.76	3.88 (3.26–4.17)	0.022 **
Achilles Tendon SWE (L) (kPa)	79.12 ± 73.22	57.76 (15.38–145.86)	22.31 ± 23.68	14.73 (11.32–17.16)	0.017 **
Achilles Tendon Thickness (R) (cm)	0.49 ± 0.06	0.49 (0.44–0.49)	0.47 ± 0.1	0.46 (0.37–0.53)	0.765 *
Achilles Tendon SWE (R) (m/s)	6.33 ± 2.29	6.7 (4.40–8.52)	4.43 ± 1.62	3.85 (3.49–4.92)	0.035 **
Achilles Tendon SWE (R) (kPa)	59.96 ± 50.06	46.37(19.70–90.91)	23.26 ± 22.21	15.17 (12.46–25.30)	0.028 **

*: Independent Sample *t*-test; **: Mann–Whitney U test.

## Data Availability

The raw data supporting the conclusions of this article will be made available by the authors upon request.
